# The Lipid Lowering and Cardioprotective Effects of *Vernonia calvoana* Ethanol Extract in Acetaminophen-Treated Rats

**DOI:** 10.3390/medicines4040090

**Published:** 2017-12-12

**Authors:** Godwin Eneji Egbung, Item Justin Atangwho, Ochuole Diana Odey, Victor Ndubuisi Ndiodimma

**Affiliations:** Department of Biochemistry, University of Calabar, P.M.B 1115, Calabar 540 Nigeria; dratangwho@gmail.com (I.J.A.); diianer12@yahoo.com (O.D.O.); ndiodimmavictor@gmail.com (V.N.N.)

**Keywords:** Acetaminophen, *Vernonia calvoana*, serum lipid indices, hypolipidemic activity and antioxidants

## Abstract

**Background:** Paracetamol overdose/abuse as a result of self-medication is a common occurrence amongst people living in low/middle income countries. The present study was designed to investigate the hypolipidemic and cardioprotective potentials of *Vernonia calvoana* (VC) ethanol extract in acetaminophen (paracetamol)-treated rats. **Methods:** Thirty-five Wistar rats weighing 100–150 g were randomly assigned into five groups of seven rats each. Groups 2–5 received high doses of paracetamol to induce liver damage, while group 1 was used as normal control. Afterwards, they were allowed to receive varying doses of VC (group 3 and 4) or vitamin E (group 5), whilst groups 1 and 2 were left untreated. The treatment period lasted for twenty one days after which sera were harvested and assayed for serum lipid indices using standard methods. **Results:** Groups 3 to 5 treated animals indicated significant decrease (*p* < 0.001) in low density lipoprotein cholesterol (LDL-c), total cholesterol (TC) and triacylglycerol (TG) levels relative to the normal and acetaminophen-treated controls, the atherogenic index showed a significant decrease (*p* < 0.001) in all treated groups compared with normal and acetaminophen-treated controls. However, the VC- and vitamin E-treated groups showed significant (*p* < 0.001) increase in high density lipoprotein cholesterol (HDL-c) relative to the controls. **Conclusions:** Data from our study suggest that ethanol leaf extract of VC possesses probable hypolipidemic and cardioprotective effects.

## 1. Introduction

Acetaminophen, also known as paracetamol, is most often classified as a mild, over-the-counter analgesic used in the treatment of pains/headaches. It is mostly intentionally abused. The drug is generally safe when taken in recommended doses, though even a very small overdose could be deleterious. In the United States of America, paracetamol overdose has been reported to account for more calls to the poison control center than an overdose of any other pharmacological substance [1]. It is the number one drug of choice in managing pains globally. However, its mechanism of action in relieving pain is yet to be fully elucidated but suggested to be implicated in a number of pain pathways [2].

Paracetamol metabolism in the liver could result in the formation of a highly toxic metabolite, *N*-acetyl-*p*-benzoquinone imine (NAPQI) by the cytochrome P_450_ enzyme system [3]. Further detoxification to eliminate the metabolite is accomplished by its conjugation with glutathione, but in cases of overdose or abuse, glutathione stores are depleted resulting in the accumulation of the metabolite and eventual toxicity [3]. Abuse of the drug may lead to toxicity which could result in hepatocellular necrosis and kidney damage [4]. Oxidative stress mediated action of NAPQI accumulation has been implicated in the pathogenesis of paracetamol-induced liver and renal damage in experimental animals [4]. Paracematol abuse in third world countries has been on the increase since a greater proportion of the population tends to resort to self-medication. The non-availability of standard health facilities poses another challenge of the arbitrary use of ethno botanicals instead of synthetic drugs to treat complications of paracetamol abuse.

Medicinal plants have played an important role in the abatement of toxic substances in the human body. They also function as vital hypolipidemic agents [5]. *Vernonia calvoana* (Hook.F) is an asteraceae with local name “Ekeke leaf” by the indigenes of the central senatorial district of Cross-River State of Nigeria [6]. The plant is distributed in the upper Guinea Cameroun mountains, South-West Cameroun and South-Eastern Nigeria [7]. It is consumed by natives based on the belief that the plant remedies heart diseases, diabetes, malaria, stomach aches and can be used as a vermifuge [6]. Igile et al. and Egbung et al. [6,8], respectively, reported that the leaves and the inflorescents of *Vernonia calvoana* contained flavonoids in appreciable amounts thus responsible for its antioxidant properties. Iwara et al. [9] also reported its hepatoprotective, hypolipidemic and antidiabetic activity in Steptozotocin-induced rats. Folklore medicine and ethno botanicals application when not developed could go into extinction [10]. Most medicinal plants have outstanding therapeutic effects and are better tolerated than some synthetic drugs, and as such produce fewer allergic reactions [11]. However, a good number of these herbal formulations may exert some toxic effect as well [12].

*Vernonia amygdalina*, a member of the same genus like *Vernonia calvoana* has been exploited in the management of certain disease conditions like diabetes and obesity thus exerting anti-diabetic, anti-bacterial, anti-malarial, anti-fungal, antioxidant, hepatoprotective, and non-cytotoxic properties [13]. However, there is scanty information on the probable lipid lowering and cardio protective potentials of *Vernonia calvoana* extracts in acetaminophen-treated Wistar rats. This study was therefore designed to investigate this information gap and recommend its use as alternative therapy in acetaminophen toxicities.

## 2. Materials and Methods

### 2.1. Procurement of Leaves, Rat Chow and Acetaminophen

Fresh *Vernonia calvoana* leaves were purchased from a local market in Ugep town in Yakurr Local Government Area of Cross-River State, Nigeria. The rat chow was bought from Pfizer Livestock Feeds, Aba, Abia State, Nigeria. The leaves were authenticated by Pastor Frank Aposeye, a botanist in the Department of Botany, University of Calabar, Calabar, Cross-River State, Nigeria, and voucher number BOT/VC/2/2015 deposited in the herbarium of the same department. Paracetamol manufactured by Emzor pharmaceuticals was purchased from Anijah Pharmacy, Etta Agbor Road, Calabar, Cross River State, Nigeria.

### 2.2. Preparation of Extract

The leaves were washed thoroughly to remove dust and other forms of dirt, and afterwards, air dried at room temperature (27 °C ± 2 °C) for seven days to remove moisture until completely dry. The dried leaves were blended to a fine powder using a dry Moulinex super blender and stored in air-tight containers. 1.5 kg of the powder was weighed using an electronic scale and afterwards soaked in 2000 mL of 98% ethanol (*v*/*v*) in a ratio of 3:4, i.e., (powder/solvent). To allow for proper mixing of the powder and the solvent, the mixture was agitated and then put in air-tight containers. The containers holding the mixtures were kept in the refrigerator at a temperature of 4 °C for 48 hr. Filtration of the mixture was accomplished first by using a cheesecloth, followed by the Whatman No. 1 filter paper (24 cm). The filtrate was concentrated using a rotary evaporator (model RE52A, Zhengzhou, China) to 10% of its original volume at a temperature of 37–40 °C. It was then concentrated to complete dryness in a water bath. The extract was afterwards refrigerated at 2–8 °C until when required for administration.

### 2.3. Experimental Animals

Thirty-five male albino Wistar rats weighing between 100 and 150 g were obtained from a disease-free stock of the animal house, Department of Zoology, University of Calabar, Calabar. The animals were acclimatized for two weeks on pelletized rat chow and water provided *ad libitum*. The experiment was conducted in accordance with the internationally accepted principles for laboratory animal use and care [14]. Permission and approval (009BC20816) for the use of the animals to carry out the study were obtained from the Faculty Animal Research Ethics Committee, Faculty of Basic Medical Sciences (FAREC-FBMS) University of Calabar on 20 August 2016. The animals were distributed randomly into five groups of seven animals each based on weight as shown in Table 1.

Hepatic damage was induced with paracetamol (2 g/kg b.w.) prepared in normal saline and administered per orals once a day to all the groups except the normal control for four days. Three days after paracetamol administration, one animal was selected at random from all the groups and sacrificed under anesthesia. Whole blood was collected, centrifuged and sera obtained used for the estimation of serum enzymes (aspartate aminotransferase, alanine aminotransferase and alkaline phosphatase) to confirm toxicity.

### 2.4. Extract and Drug Administration

The doses (200 mg/kg and 400 mg/kg) used were based on the predetermined LD_50_ values obtained using Lorke’s method [15]. The extract was diluted in normal saline, which acted as the vehicle and administered orally through gastric intubation accordingly after hepatic damage had been established. The control animals received 0.2 mL of normal saline. VC treatment lasted 21 days. The animals were fasted 12 hr overnight prior to the time of sacrifice. The animals were euthanised with chloroform and blood samples collected via cardiac puncture.

### 2.5. Preparation of Serum for Biochemical Assays

Whole blood was collected from each experimental animal through cardiac puncture and put into to sterile non heparinized sample tubes which were allowed to stand for 2–4 hr before centrifugation. The serum was carefully taken out using a syringe and needle down the side of the tube, leaving the clot behind. The serum gotten was subjected to further separation by centrifugation, using an MSE table top centrifuge (Buckinghamshire, England), set at 8000 rpm (revolutions per minute) for 15 min to ensure clear supernatant devoid of traces of red cells The serum samples collected were stored in a refrigerator at 4 °C for subsequent biochemical assays.

### 2.6. Assay of Selected Lipid Parameters

Triacylglycerol (TG), total cholesterol (TC), and high density lipoprotein cholesterol (HDL-c) were determined with analytical kits from Randox laboratories Ltd. (Admore Diamond Road, Crumlin, Co., Antrim, UK). Very low density lipoprotein cholesterol (VLDL-C), low density lipoprotein—cholesterol (LDL-c) and atherogenic index were estimated by modification of Friedewald formula as described by [16].The assays were conducted according to the manufacturer’s instructions.

### 2.7. Statistical Analysis

Data obtained were expressed as mean ± standard error of mean (SEM). One-way analysis of variance was used to determine the differences between means, followed by posthoc multiple comparisons. Data were considered significant at *p* < 0.05. Computer software SPSS version 17.0 and Microsoft Excel (2007 version) analyzer were used for analysis.

## 3. Results

**Figure 1:**
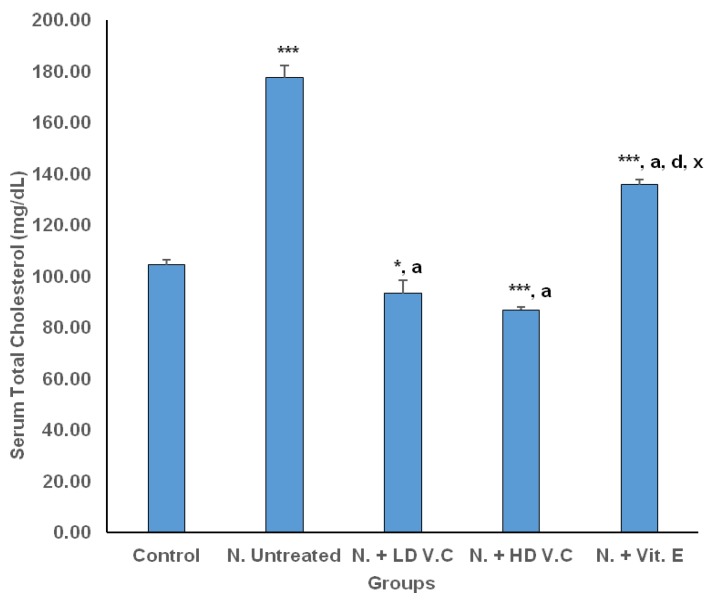
Effect of *V. calvoana* extract on serum total cholesterol in paracetamol-treated rats. Values are presented as mean ± SEM, *n* = 5. * *p* < 0.05, *** *p* < 0.001 vs. control, a = *p* < 0.001 vs. N. untreated, d = *p* < 0.001 vs. N + LD VC, x = *p* < 0.001 vs. N + HD VC. Where N = hepatotoxic.

**Figure 2:**
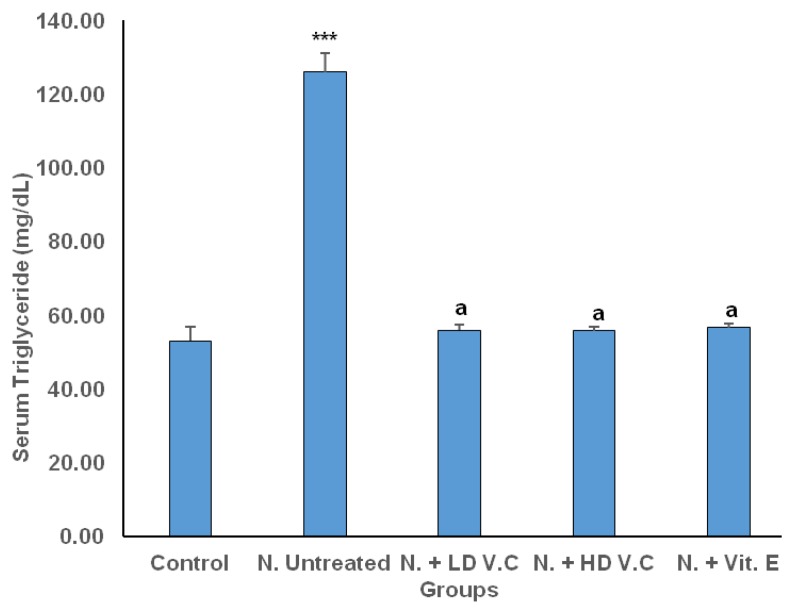
Effect of *V. calvoana* extract on serum triacylglycerol in paracetamol treated rats. Values are presented as mean ± SEM, *n* = 5. *** *p* < 0.001 vs. control, a = *p* < 0.001 vs. N. untreated. Where N = hepatotoxic.

**Figure 3:**
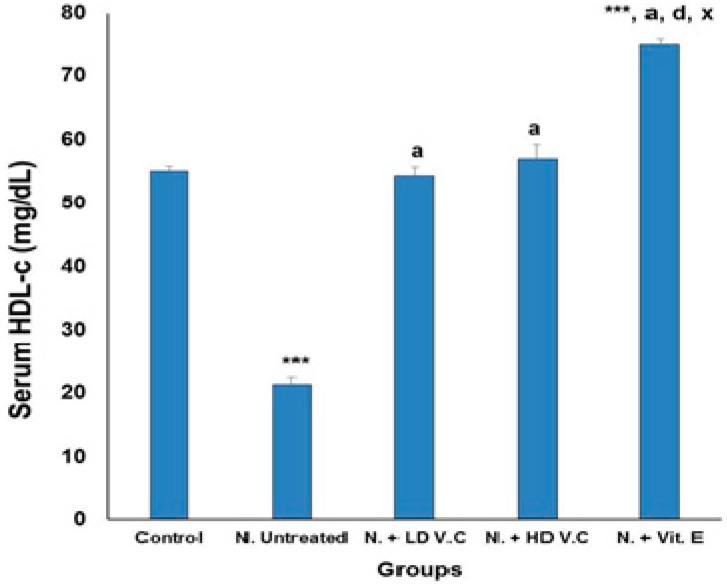
Effect of *V. calvoana* extract on serum high density lipoprotein cholesterol in paracetamol treated rats. Values are presented as mean ± SEM, *n* = 5. *** *p* < 0.001 vs. control, a = *p* < 0.001 vs. N. untreated, d = *p* < 0.001 vs. N. + LD V.C., x = *p* < 0.001 vs. N. + HD V.C. Where N = hepatotoxic.

**Figure 4:**
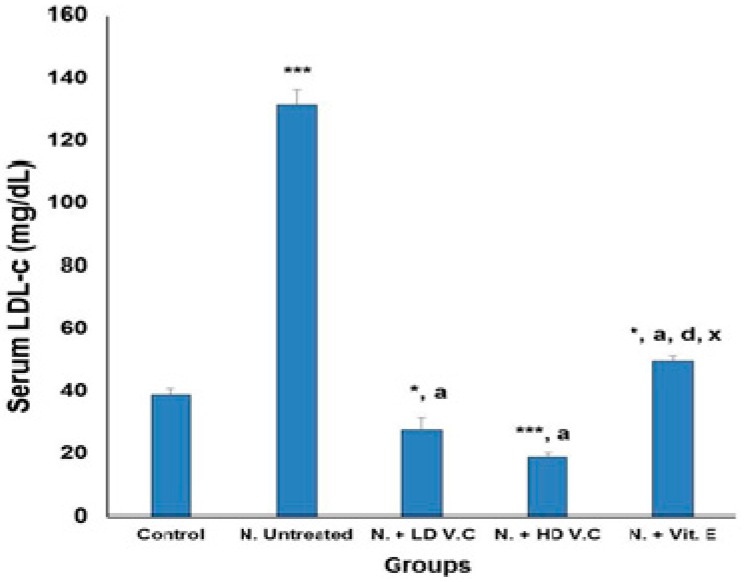
Effect of *V. calvoana* extract on serum low density lipoprotein cholesterol (LDL-c) in paracetamol treated rats. Values are presented as mean ± SEM, *n* = 5. * *p* < 0.05, *** *p* < 0.001 vs. control, a = *p* < 0.001 vs. N. untreated, d = *p* < 0.001 vs. N. + LD V.C., x = *p* < 0.001 vs. N. + HD V.C. Where N = hepatotoxic.

**Figure 5:**
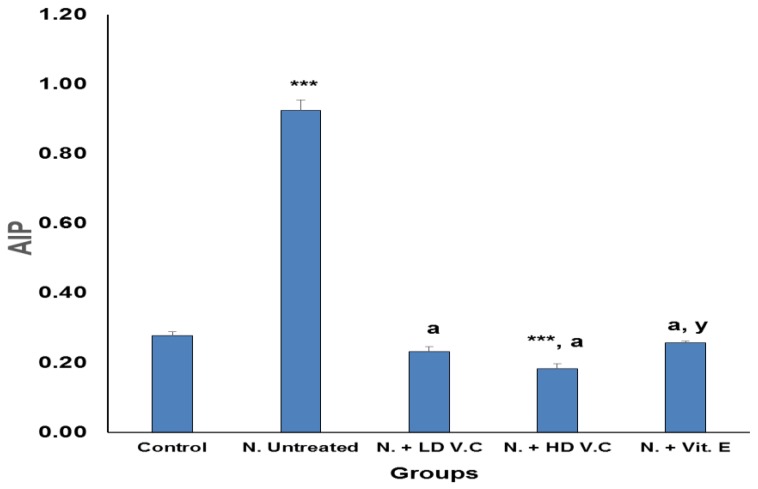
Effect of *V. calvoana* extract on the atherogenic index in paracetamol treated rats. Values are presented as mean ± SEM, *n* = 5. *** *p* < 0.001 vs. control, a = *p* < 0.001 vs. N. untreated, y = *p* < 0.01 vs. N. + HD V.C. Where N = hepatotoxic.

Results of the effects of *V. calvoana* leaf extract on some selected lipid biomarkers in paracetamol-treated rats are presented in Figure 1, Figure 2, Figure 3, Figure 4 and Figure 5.

### 3.1. Effects of V. calvoana on Total Cholesterol (TC) Concentration 

The mean total cholesterol values for the different experimental groups (control group, hepatotoxic untreated group, 400 mg/kg bwt *V. calvoana* treated group, 200 mg/kg bwt *V. calvoana* treated groups and vitamin E-treated group namely groups 1, 2, 3, 4 and 5 respectively) are 104.45 ± 1.86, 177.52 ± 4.72, 93.27 ± 5.08, 86.80 ± 1.32 and 135.78 ± 1.95 respectively. The mean total cholesterol value for the hepatotoxic untreated group significantly increased at *p* < 0.001 when compared to the normal control group. The group treated with 200 mg/kg bwt of the extract had a significantly lower TC value at *p* < 0.005 compared to the normal control and a significantly lower value at *p* < 0.001 when compared to the hepatotoxic untreated group. The group treated with 400 mg/kg bwt of the extract showed a much more reduced value significant at *p* < 0.001 when compared with the normal control and the hepatotoxic untreated group. The vitamin E-treated group significantly reduced at *p* < 0.001 when compared with the hepatotoxic untreated group, but had a higher value than the normal control and the *V. calvoana* treated groups, significant at *p* < 0.001 respectively. The result obtained above is represented in Figure 1.

### 3.2. Effect of V. calvoana on Triacylglycerol Concentration

Triacylglycerol mean values for the groups are 53.09 ± 3.88, 125.97 ± 5.12, 55.95 ± 1.51, 55.75 ± 1.15, and 56.62 ± 1.24 respectively. The mean value for group 2 was significantly higher at *p* < 0.001 when compared with the normal control. The *V. calvoana* treated groups (200 mg/kg b.wt and 400 mg/kg b.wt) and vitamin E-treated showed significantly lower values than the untreated hepatotoxic group (*p* < 0.001). The result as presented in Figure 2.

### 3.3. Effect of Treatment on High-Density Lipoprotein Cholesterol

The high density lipoprotein cholesterol (HDL-c) values for the groups are 55.13 ± 0.71, 21.23 ± 1.21, 54.38 ± 1.38, 57.02 ± 2.23 and 75.16 ± 0.82 respectively. The mean HDL-c value of the hepatotoxic untreated group was significantly (*p* < 0.001) lower than the normal control group. The *V*. *calvoana* treated groups (200 mg/kg b.wt and 400 mg/kg b.wt) have significantly higher HDL-c values than the untreated hepatotoxic group at *p* < 0.001. The vitamin E-treated group has a significantly higher value at *p* < 0.001, compared to the normal control, untreated hepatotoxic group and the *V. calvoana* treated groups. The result as presented in Figure 3.

### 3.4. Effect of Treatment on the Low-Density Lipoprotein Cholesterol

The mean LDL-c values for all the groups are 38.70 ± 2.12, 131.09 ± 5.07, 27.70 ± 4.04, 18.63 ± 1.43 and 49.30 ± 1.68. Group 2 has a significantly higher value at *p* < 0.001 when compared to the normal control. Mean LDL-c value for group 3 showed a significant decrease at *p* < 0.05 and *p* < 0.001 compared to the normal control (group 1) and group 2 respectively. Compared to group 1 and 2, group 4 showed a significant decrease at *p* < 0.001 respectively, while the mean value for group 5 was significantly lower at *p* < 0.05 compared to group 1. However, compared to group 3 and 4, the mean LDL-c value of group 5 was significantly higher at *p* < 0.001. The mean value of group 5 dropped significantly (*p* < 0.001) compared to group 2. The result as presented in Figure 4.

### 3.5. Effect of Treatment on Atherogenic Index

Figure 5 shows that group 2 has a significantly (*p* < 0.001) higher atherogenic index of plasma compared to the normal control (group 1). Group 3 has a significantly lower value at *p* < 0.001 compared to group 2. Similarly, group 4 has a significantly lower atherogenic index compared to group 2 at *p* < 0.001, and a lower value also compared to group 2 at *p* < 0.001. However, group 5 has a significantly lower value as well compared to group 2 at *p* < 0.001, but a significantly higher value than group 4 at *p* < 0.01.

## 4. Discussion

The prevalence of drug abuse as a result of overdose has been increasing, especially in economically deprived communities in the developing countries where there is little or no access to standard health facilities and the majority of the population resort to self-medication [17]. In this study, we reported the effect of *V. calvoana* leaf extract on some selected lipid biomarkers in paracetamol-treated Wistar rats. The 400 mg/kg body weight extracts of *V. calvoana* exert more potent lipid lowering activity which is similar to the pattern in a study by Iwara et al. [9]. Acetaminophen poisoning may be due to ingestion of excessive/repeated or too frequent doses [18]. Obu et al. [19] reported that most administration of paracetamol to children was done through self-prescription with possible tendency of abuse. The paracetamol treated rat model used was to mimic a situation where persons especially involved in unskilled labor practice will walk into patent medicine stores to purchase paracetamol for treating pains due to a hard day’s labor at construction/building sites. The trend continues for about three to four days in a week. *V. calvoana* is safe because its dose at above 5000 mg/kg body weight in mice showed no toxicity from our preliminary studies. The animal model groups treated with 200 mg/kg b.wt and 400 mg/kg b.wt of the *V. calvoana* (groups 3 and 4) showed significantly lower LDL-C, TC and atherogenic index, as well as increased HDL-c levels. This finding is in agreement with the report of [9], where the extract was administered to diabetic rats and it exhibited a lipid lowering effect. Oxidative stress conditions will often trigger lipid peroxidation as represented by increased levels of malondialdehyde concentrations. The metabolite of acetaminophen has been known to be a depleting agent of the glutathione pool, the body’s antioxidants defense system. The administration of ethanol extracts of *V. calvoana* mimics the replenishing potentials of glutathione, thus, preventing free radical generation following overdose of paracetamol.

The increased HDL-c levels noticed in groups 3 and 4 indicates a possibly lower risk of developing coronary heart diseases and other related cardiovascular events. Nichols et al. [20] reported that a moderate rise in HDL-c levels, resulting from the use of statin drugs, has been linked to a corresponding decline in the risk of developing coronary atherosclerosis. Stocker et al. [21] also reported that lowering LDL-c was a more effective method of reducing the risk of developing cardiovascular diseases than surgical methods. However, the abundance of antioxidants in the plant (*V. calvoana*) could prevent the oxidation of LDL-c within the blood vessels. It is a known fact that LDL-c is virtually harmless till it is oxidized by reactive species in the blood vessels, resulting in atherosclerosis [22]. The reduced atherogenic indices of the extract-treated groups implies a decreased risk of developing atherosclerosis since the atherogenic index of plasma is considered a reliable indicator of the onset of cardiovascular events [23,24]. Our finding is in agreement with the hypolipidemic effect of *V. calvoana* extracts in a diabetic rat model as reported by Iwara et al. [9] which implicated flavonoids and other bioactive principles for the effects. They are also in agreement with the report of Johnson et al. [25] where *V. amygdalina* decreased levels of MDA and prevented peroxidation of lipids in PC-3 cells.

## 5. Conclusions

From the results obtained in this study, it is safe to further confirm that the ethanolic leaf extract of *V. calvoana* possesses a lipid-lowering and cardioprotective effects and is a candidate in the management of paracetamol-induced toxicities, however, further studies on the molecular mechanism underlying the effect of *V. calvoana* on functional lipids is being investigated in our laboratory.

## Figures and Tables

**Table 1:** Experimental treatment groups.

Group	Number of Animals	Treatment
Group 1	7	Normal saline
Group 2	7	2 g/kg paracetamol only
Group 3	7	2 g/kg paracetamol + 200 mg/kg b.w. VC
Group 4	7	2 g/kg paracetamol + 400 mg/kg b.w. VC
Group 5	7	2 g/kg paracetamol + 100 mg/kg b.w. Vit. E

VC = Extract of *Vernonia calvoana*; Vit. E = Vitamin E; b.w. = body weight.
